# Relationships Between Rapid Eye Movement Sleep Behavior Disorder and Parkinson’s Disease: Indication from Gut Microbiota Alterations

**DOI:** 10.14336/AD.2023.0518

**Published:** 2024-02-01

**Authors:** Pingchen Zhang, Pei Huang, Yuanyuan Li, Juanjuan Du, Ningdi Luo, Yixi He, Jin Liu, Guiying He, Shishuang Cui, Weishan Zhang, Gen Li, Xin Shen, Liu Jun, Shengdi Chen

**Affiliations:** ^1^Department of Neurology and Institute of Neurology, Ruijin Hospital, Shanghai Jiao Tong University School of Medicine, Shanghai 200025, China.; ^2^Lab for Translational Research of Neurodegenerative Diseases, Shanghai Institute for Advanced Immunochemical Studies (SIAIS), Shanghai Tech University, Shanghai 201210, China

**Keywords:** Rapid eye movement sleep behavior disorder, Parkinson’s disease;, Gut microbiota, 16S rRNA

## Abstract

Rapid eye movement sleep behavior disorder (RBD) has a close relationship with Parkinson’s disease (PD) and was even regarded as the most reliable hallmark of prodromal PD. RBD might have similar changes in gut dysbiosis to PD, but the relationship between RBD and PD in gut microbial alterations is rarely studied. In this study, we aim to investigate whether there were consistent changes between RBD and PD in gut microbiota, and found some specific biomarkers in RBD that might indicate phenoconversion to PD. Alpha-diversity showed no remarkable difference and beta-diversity showed significant differences based on the unweighted (R = 0.035, *P* = 0.037) and weighted (R = 0.0045, *P* = 0.008) UniFrac analysis among idiopathic RBD (iRBD), PD with RBD, PD without RBD and normal controls (NC). Enterotype distribution indicated iRBD, PD with RBD and PD without RBD were *Ruminococcus*-dominant while NC were *Bacteroides*-dominant. 7 genera (4 increased: *Aerococcus*, *Eubacterium*, *Gordonibacter* and *Stenotrophomonas*, 3 decreased: *Butyricicoccus*, *Faecalibacterium* and *Haemophilus*) were consistently changed in iRBD and PD with RBD. Among them, 4 genera (*Aerococcus*, *Eubacterium*, *Butyricicoccus*, *Faecalibacterium*) remained distinctive in the comparison between PD with RBD and PD without RBD. Through clinical correlation analysis, *Butyricicoccus* and *Faecalibacterium* were found negatively correlated with the severity of RBD (RBD-HK). Functional analysis showed iRBD had similarly increased staurosporine biosynthesis to PD with RBD. Our study indicates that RBD had similar gut microbial changes to PD. Decreased *Butyricicoccus* and *Faecalibacterium* might be potential hallmarks of phenoconversion of RBD to PD.

## INTRODUCTION

Parkinson's disease (PD) is characterized by cardinal motor symptoms including bradykinesia, resting tremor and rigidity [1], while various non-motor symptoms (NMS) often precede the onset of motor symptoms. NMS have attracted plenty of attention as clues for identifying prodromal stage of PD, which is a key point to predict the conversion to PD and administer disease-modifying interventions [2]. These prodromal NMS include olfactory loss, constipation and sleep disorders. Among sleep disorders, [rapid eye movement (REM) sleep behavior disorder] (RBD) is regarded as the most reliable hallmark of prodromal PD [3,4]. RBD is manifested by dream-enacting behaviors and nightmares associated with REM sleep without common muscle atonia [5]. The majority of idiopathic RBD (iRBD) patients have a high likelihood ratio to develop PD. The largest multi-center study of 1280 polysomnographically (PSG)-diagnosed RBD subjects reported their phenoconversion to overt neurodegenerative syndrome of 6.3% per year, with more than half developed to PD [6]. Progressive loss of presynaptic dopamine terminals in the striatum was proved to act as a promising neuroimaging biomarker in iRBD to aid the prediction of phenoconversion to PD [7,8].

The above evidence indicates that RBD has a close relationship with PD and might even be the prodromal stage of PD based on the clinical phenoconversion and neuroimaging presentation. Recent studies might strengthen the level of evidence from the point view of gut dysbiosis. First, among those RBD patients who finally converted to PD, constipation acted as a clinical predictor for the phenoconversion [8]. Second, gut dysbiosis has been considered to play an important role in the onset of PD. The contribution of microbiota-gut-brain axis to PD pathophysiology was reported by several studies [9,10]. Gut dysbiosis can lead to local or systemic inflammation, which may facilitate PD pathophysiology. Aberrant alpha-synuclein translocates from the gut to the brain via enteric nerves while neuroinflammation is activated through the transportation of bacterial products and proinflammatory cytokines from gut via circulation [11]. A recent longitudinal study found PD patients could be divided into two patterns based on the appearance order of symptoms, i.e. body-first and brain-first. Body-first cases usually began with gut dysbiosis, and this subtype of PD patients mostly had a prodromal phase of RBD [12]. We may conclude that gut dysbiosis is more expected to be observed in PD with RBD patients. As gut microbiota plays a crucial role in gastrointestinal homeostasis, it’s worthwhile to explore microbial changes in PD and RBD and find the underlying relationships between them.

To date, there existed two studies of gut microbiota targeted iRBD and PD patients from Germany [13] and Japan [14], and one large cohort study in Germany [15] to investigate the association of microbial composition and risk factors in PD, including possible RBD. The former Germany group collected the nasal and gut microbiome from 76 PD patients, 21 iRBD patients and 78 normal controls (NC) and assessed by 16S and 18S ribosomal RNA amplicon sequencing and metagenome analysis based on operational taxonomic unit (OTU) level. They found more than 75% of the differentially abundant OTUs in the comparisons of PD or iRBD versus NC showed qualitatively similar changes compared to NC, but most of them did not remain statistically significant. They also found the increase of *Akkermansia* and *Provotella* in both iRBD and PD with RBD when compared with PD without RBD, but this trend did not remain statistically significant in the comparison of iRBD and PD [13]. Another study from Japan analyzed gut microbiota in 26 iRBD patients and 137 controls by 16S rRNA sequencing in their data set and meta-analyzed with the abovementioned Germany dataset. They also demonstrated genus *Akkermansia* and family *Akkermansiaceae* were consistently increased in both iRBD and PD [14]. In addition, a study in Germany enrolled 666 individuals to detect the gut microbiome signatures of risk and prodromal markers in PD by 16S rRNA sequencing. This study found a relative decrease in *Faecalicoccus* and *Victivallis*, and a relative increase in the abundance of *Haemophilus* related to possible RBD according to RBD screening questionnaire [15]. None of them considered the interference of anti-PD medication and nor did they investigate exactly the difference between PD with RBD and PD without RBD. In addition, these three relevant studies only adopted relative abundance analysis, possibly resulting in inappropriate interpretation of quantitative microbiota profiling [16].

In this study, we enrolled *de novo* PD patients, iRBD patients and NC. PD patients were further divided into two subtypes: PD with RBD and PD without RBD. We intended to identify similar gut microbiota alterations in PD with RBD and iRBD, which could possibly act as potential microbial biomarkers for the transition from iRBD to PD. Secondly, we analyzed the differential gut microbiota between PD with RBD and PD without RBD. This may help provide microbiological explanations for the clinical differences between these two PD subtypes. For those genera which shared consistent changes in PD with RBD and iRBD, and simultaneously remained specific in PD with RBD rather than PD without RBD, we considered them as specific to RBD and they could act as potential hallmarks of phenoconversion from iRBD to PD. Finally, the correlation between these RBD-specific genera and RBD clinical features was analyzed to confirm their clinical relationships to RBD.

## MATERIALS AND METHODS

### Study Subjects

*30 de novo* PD patients with RBD, 64 *de novo* PD patients without RBD, 35 iRBD patients and 60 matched NC from outpatient clinic of Movement Disorders Center in Ruijin Hospital affiliated to Shanghai Jiao Tong University School of Medicine were enrolled in our study over the past five years. This study was approved by the Institutional Review Board of Ruijin Hospital affiliated to Shanghai Jiao Tong University School of Medicine (No. 2018-243). Written informed consents were obtained from all participants according to the Declaration of Helsinki. All iRBD patients were diagnosed by PSG according to the consensus criteria of the International RBD Study group [17] with no signs for neurodegenerative disorders. All PD patients met the MDS criteria [18] and they did not take any anti-PD medication before the fecal sample collection. RBD in PD was diagnosed when REM sleep behavior disorder questionnaire-Hong Kong (RBD-HK) score superior to 17 [19]. NC matched by age, gender and body mass index (BMI) were enrolled simultaneously. Exclusion criteria included vegetarian or malnutrition, history of chronic gastrointestinal disorder or gastrointestinal surgery, regular consummation of yogurt, and use of 19 medications which were reported to have significant association with microbiota [20]. All the medications taken by the subjects were summarized. (Supplementary Table. 1)

### Clinical Evaluation

All subjects provided information of medical history, weight and height for the calculation of BMI, and accepted neurological examination and clinical assessment, such as Hamilton Anxiety Scale (HAMA) [21] for anxiety, Hamilton Depression Scale-17 items (HAMD-17) [22] for depression, Wexner constipation score [23] and Bristol stool scale [24] for constipation severity, Mini Mental State Examination (MMSE) [25] and Montreal Cognitive Assessment (MoCA) [26] for cognition. The Scale for Outcomes in Parkinson’s disease for Autonomic Symptoms (SCOPA-AUT) [27] and the Non-Motor Symptoms Scale (NMSS) [28] were interrogated for iRBD and PD patients for autonomic dysfunction, while RBD-HK and Parkinson's disease sleep scale (PDSS) [29] were assessed for sleep quality and the 16-item Sniffin' Sticks test (SS-16) [30] tested for odor identification. The Movement Disorder Society sponsored version of the Unified Parkinson’s Disease Rating Scale (MDS-UPDRS) and the Hoehn and Yahr stage (H-Y stage) were examined for PD patients.

### Fecal Sample Collection and Sequencing

Consistent with our previous study [31], fecal samples were collected at home or directly after consultation by using the containers we provided. The containers were transferred on ice and frozen at -80°C upon receipt. Microbial DNA extraction was based on 200 mg samples and further purified using QIAamp® Fast DNA Stool Mini Kit (Qiagen, Straße, Hilden, Germany, Cat# 51504) following the manufacturer’s instructions. The high variable V3-V4 regions of 16S rRNA were amplified with primers 357F(5'-ACTCCTACGGRAGGCAGCAG-3’) and 806R(5'-GGACTACHVGGGTWTCTAAT-3') and Phusion® High-Fidelity PCR kit (New England Biolabs, Ipswich, MA, United States Cat# E0553L) in a 50μL reaction template. PCR conditions consisted of initial denaturation at 94 °C for 2 min, followed by 25 cycles of denaturation at 94 °C for 30 s, annealing at 56 °C for 30 s and extension at 72 °C for 30 s, with a final extension of 72 °C for 5 min. The barcoded PCR products were purified using a DNA gel extraction kit (Axygen, Silicon Valley, SFO, USA, Cat# AP-GX-250G) and quantified using the FTC -3000 TM real-time PCR (Funglyn, Minhang, Shanghai, China). The PCR products from different samples were mixed at equal ratios. The second step PCR with dual 8bp barcodes was used for multiplexing. Eight cycle PCR reactions were used to incorporate two unique barcodes to either end of the amplicons. Cycling conditions consisted of one cycle of 94 °C for 3 min, followed by eight cycles of 94 °C for 30 s, 56 °C for 30 s and 72 °C for 30 s, followed by a final extension cycle of 72 °C for 5 min. The purified library was then sequenced by 2×250 bp paired-end sequencing on the Novaseq platform using Novaseq 6000 SP 500 Cycle Reagent Kit (Illumina, San Diego, CA, USA).

Meanwhile, quantification of total bacteria load was performed by quantitative real-time PCR (qRCR) with TaKaRa® SYBR PremixTaq kit (Takara, Shiga, Japan, Cat# RR420A) by triple replicates for each sample with a FTC-3000TM Real-Time Quantitative Thermal Cycler (Funglyn, Minhang, Shanghai, China). The thermal cycling conditions started with pre-denaturation at 95°C for 30s, denaturation at 95°C for 10s, annealing at 55°C for 30s, extension at 72°C for 30s (40 cycles), and final extension at 72°C. Standard curves were generated with serial dilutions of the plasmid with pMD18-T vector (Takara, Shiga, Japan, Cat# 6011), ranging from 10^7^ to 10^12^ target gene copies µl^-1^.

### Bioinformatic Statistical Analysis

Continuous variables were presented as mean±standard deviation (SD), and categorical variables were presented as percentages and numbers. Shapiro-Wilk test was used to test the normality of the distribution and Levene test was to assess the homogeneity of variance in different groups. Since almost all of our continuous variables were either non-normally distributed or with heterogeneity of variance (Supplementary Table. 2-7), we adopted Kruskal-Wallis (K-W) rank-sum test among more than two groups and Wilcoxon rank-sum test between two groups.

Demographic and clinical characteristics of all subjects were compared by K-W/Wilcoxon test for continuous variables and chi-squared test for categorical variables.

The 16S sequences were analyzed by using a combination of software Trimmomatic (version 0.35) [32], Flash (version 1.2.11) [33], UPARSE (version v8.1.1756) [34], mothur (version 1.33.3) [35] and R (version 3.6.3) [36]. The raw 16s rRNA gene data were clustered into operational taxonomic units (OTUs) at 97% identity using UPARSE. Taxonomy was assigned according to Sliva 128 reference database. Then the relative abundance obtained from 16S rRNA sequencing was quantified to get absolute abundance (copies/gram) by multiplying the total bacterial load performed by qPCR.

Diversity and enterotype in gut bacterial community were analyzed based on relative abundance. Alpha diversity analysis was performed using Chao index for species richness, Shannon and Simpson indices for species diversity by K-W test. Beta diversity analysis between groups was calculated by analysis of similarities (ANOSIM) [37] based on weighted and unweighted UniFrac distance matrices. Meanwhile, enterotype features were examined by dividing into 3 clusters based on the Jensen-Shannon divergence [38] and the distribution was measured by chi-squared test.

The genus level was selected for further analysis and only the consistent results based on relative and absolute abundances were retained. Linear discriminant analysis Effect Size (LEfSE) [39] analysis was used for between-group comparisons with an alpha cutoff of 0.05 and an effect size cutoff of 2.0. Spearman rank-correlation analysis was applied to explore the relationships between RBD-related clinical features (RBD-HK and RBD disease duration) and specific microbiota taxa, which had consistent changes in iRBD and PD with RBD selected in the LEfSE analysis. Ridge regression analysis was then performed after Spearman correlation analysis to avoid the confounding effect, after adjusting for sex, age, BMI, smoking, alcohol drinking and diabetes.

Updated Phylogenetic Investigation of Communities by Reconstruction of Unobserved States (PICRUSt2) [40] was used to predict the changes of functional gene composition in fecal microbiota in Kyoto Encyclopedia of Genes and Genomes (KEGG). Wilcoxon test was used for the differential analysis between groups at level 3. *P* < 0.05 was considered statistically significant.

**Table 1:** Demographics and clinical characteristics of the subjects.

Variables	iRBD (n=35)	PD with RBD (n=30)	PD without RBD (n=64)	NC (n=60)	*P* value
Age (y)^a^	64.63±9.69	65.63±7.96	62.31±10.87	62.60±6.05	0.105
Male %^b^	74.2%	60.0%	60.9%	48.3%	0.097
BMI (kg/m^2)^a^	23.99±2.43	23.64±2.78	23.39±2.26	23.73±3.13	0.652
Smoke (±)^b^	9.3%	16.7%	29.7%	26.7%	0.090
Alcohol (±)^b^	11.4%	23.3%	31.2%	20.0%	0.142
Diabetes (±)^b^	8.6%	13.3%	20.3%	8.3%	0.194
MMSE^a^	29.14±1.14	27.70±2.38	28.31±1.85	28.10±1.70	0.013^*^
MoCA^a^	26.29±3.74	24.80±2.73	24.97±3.82	27.35±1.51	<0.001^***^
HAMD-17^a^	4.54±4.17	5.43±6.89	4.55±4.70	1.13±1.83	<0.001^***^
HAMA^a^	5.06±3.76	4.93±6.1	4.03±4.25	1.57±2.09	<0.001^***^
Bristol^a^	3.09±1.12	4.30±1.44	4.28±1.17	4.15±0.99	<0.001^***^
Wexner^a^	6.83±4.44	5.10±4.56	2.45±2.98	0.90±1.66	<0.001^***^
PD disease duration^a^	/	2.40±2.28	2.41±2.16	/	0.801
RBD disease duration^a^	5.57±5.23	3.12±2.81	/	/	0.004^**^
RBD-HK^a^	40.8±18.7	29.83±10.48	2.19±3.46	/	<0.001^***^
SCOPA-AUT^a^	8.69±9.85	6.87±5.56	4.83±5.76	/	0.048^*^
NMSS^a^	14.37±14.28	20.07±26.4	11.20±13.84	/	0.032^*^
SS-16^a^	8.20±4.07	6.63±3.73	7.86±3.86	/	0.241
PDSS^a^	133.46±10.63	130.73±15.26	136.20±13.19	/	0.066
H-Y stage (1.0/1.5/2.0/2.5) ^b^	/	7/7/12/4	26/12/19/7	/	0.432
MDS-UPDRS^a^	/	42.17±18.77	32.66±19.18	/	0.010^*^
MDS-UPDRS III^a^	/	27.80±13.38	22.16±13.45	/	0.026^*^

BMI, body mass index; HAMA, Hamilton Anxiety Scale; HAMD-17, Hamilton Depression Scale-17 items; H-Y stage, Hoehn and Yahr stage; MDS-UPDRS, Movement Disorder Society sponsored version of the Unified Parkinson’s Disease Rating Scale; MMSE, Mini Mental State Examination; MoCA, Montreal Cognitive Assessment; NMSS, Non-Motor Symptoms Scale; RBD, rapid eye movement behavior disorder; PD, Parkinson’s disease; PDSS, Parkinson's disease sleep scale; RBD-HK, REM sleep behavior disorder questionnaire-Hong Kong; SCOPA-AUT, Scale for Outcomes in Parkinson’s disease for Autonomic Symptoms; SS-16, 16-item Sniffin' Sticks test.

a Data were shown as mean ± standard deviation (SD), compared by Kruskal-Wallis(K-W) rank-sum test or Wilcoxon rank-sum test.

b Data were shown as percentage% , compared by chi-squared test.

* *P*<0.05,

** *P*< 0.01,

*** *P*<0.001.

**Figure 1.**
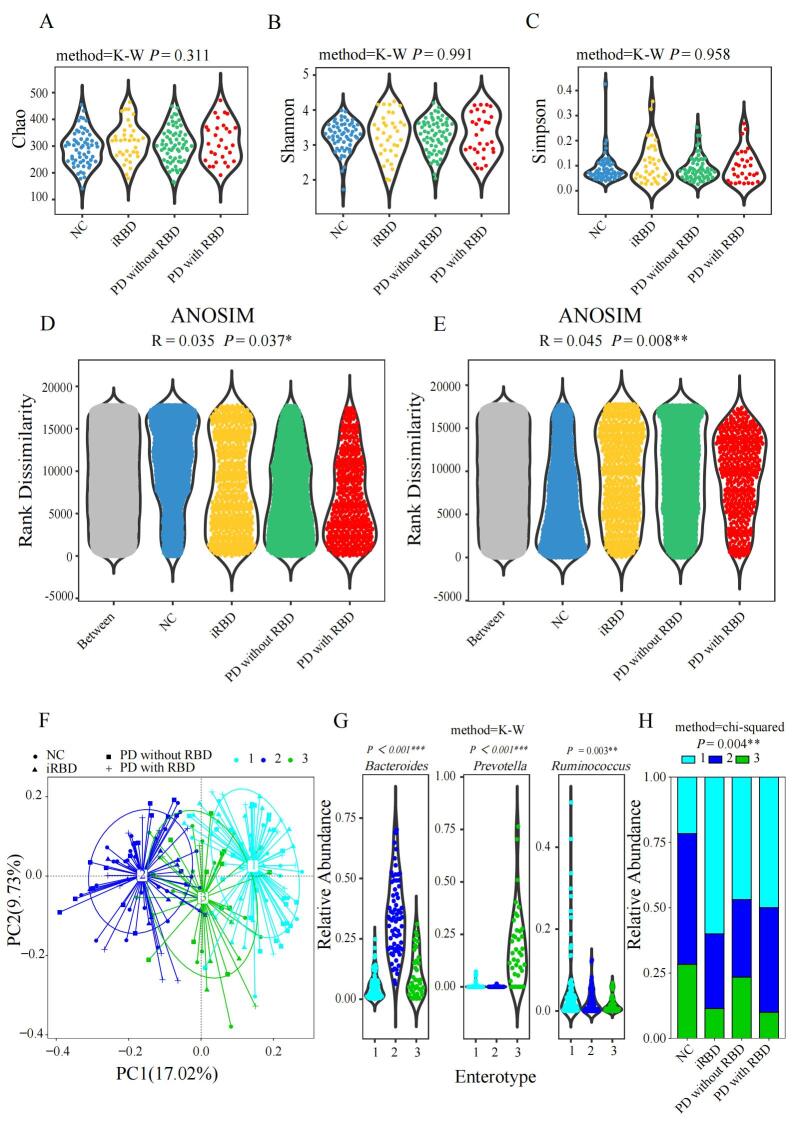
**Between-group comparisons of alpha diversity, beta diversity and enterotype distribution**. (**A**) Chao index of alpha diversity of gut microbiota. (**B**) Shannon index of alpha diversity of gut microbiota. (**C**) Simpson index of alpha diversity of gut microbiota. (**D**) Beta diversity in unweighted Unifrac analysis by analysis of similarities (ANOSIM). (**E**) Beta diversity in weighted Unifrac analysis by ANOSIM. (**F**) Samples clustered into 3 enterotypes (dark blue-*Bacteriodes*, green-*Prevotella* and light blue-*Ruminococcus*) by principal co-ordinates analysis (PCoA, two-dimensional). (**G**) Relative abundances of the top genera in each enterotype: dark blue represented *Bacteriodes*, green represented *Prevotella* and light blue represented *Ruminococcus.* (H) Enterotype distribution in NC, iRBD, PD without RBD and PD with RBD. Samples were presented as dots in each chart (NC, n = 60; iRBD, n = 35; PD without RBD, n = 64; PD with RBD, n = 30). Data were measured by Kruskal-Wallis (K-W) test for alpha diversity and enterotype composition, ANOSIM for beta diversity, and chi-squared test for enterotype distribution. *P<0.05, **P<0.01, *** *P*<0.001.

## RESULTS

### Demographics and Clinical Characteristics of the subjects

The demographics and clinical characteristics of iRBD, PD with RBD, PD without RBD and NC were summarized (Table. 1). Among these 4 groups (K-W), there were no significant differences in age, gender, BMI, diabetes, as well as daily habits of smoke, alcohol (*P ≥* 0.090). PD with RBD had the lowest average score in MMSE (*P* = 0.013) and MoCA (*P* <0.001) while they had the highest score in HAMD-17 (*P* <0.001). In addition, iRBD showed the lowest Bristol score (*P* <0.001) and the highest score in Wexner (*P* <0.001) and HAMA (*P* <0.001). In the comparison between PD with RBD and PD without RBD (Wilcoxon), those with RBD exhibited higher scores in Wexner (*P* = 0.010), SCOPA-AUT score (*P* = 0.045), NMSS (*P* = 0.010), total MDS-UPDRS (*P* = 0.010), and MDS-UPDRS III (*P* = 0.026).

**Table 2:** Spearman’s correlation for specific taxa in both iRBD and PD with RBD at genus level and RBD-related clinical features.

Genus	RBD-related clinical features	Relative Correlation		Absolute Correlation	
		Coefficient	*P* value	Coefficient	*P* value
*Butyricicoccus*	RBD-HK	-0.19	0.029^*^	-0.25	0.004^**^
*Faecalibacterium*	RBD-HK	-0.20	0.025^*^	-0.22	0.012^*^
*Stenotrophomonas*	RBD disease duration	0.38	0.002^**^	0.44	<0.001^***^

RBD, rapid eye movement behavior disorder; RBD-HK, REM sleep behavior disorder questionnaire-Hong Kong. iRBD, n = 35; PD without RBD, n = 64; PD with RBD, n = 30.

* P<0.05,

** P< 0.01,

*** P<0.001.

### Microbial Diversity among Groups

The indexes including Chao (*P* = 0.311), Shannon (*P* = 0.991), Simpson (*P* = 0.958) of alpha-diversity (Fig. 1A-C) showed no remarkable difference among the four groups. However, significant differences were found in beta-diversity based on the unweighted (ANOSIM, R = 0.035, *P* = 0.037, Fig. 1D) and weighted analysis (ANOSIM, R = 0.045, *P* = 0.008, Fig. 1E) UniFrac analysis, especially for the difference between NC and PD with RBD. Enterotype distribution among four groups showed a significant difference (chi-squared, *P* = 0.004, Fig. 1F-H), as NC were rich in *Bacteroides*-dominant enterotype, and they had a lower percentage of *Ruminococcus*-enriched enterotype. The difference among iRBD, PD with RBD and PD without RBD was not significant (chi-squared, *P* = 0.323).

### Between-group Comparison of Microbial Abundant Taxa

**Figure 2.**
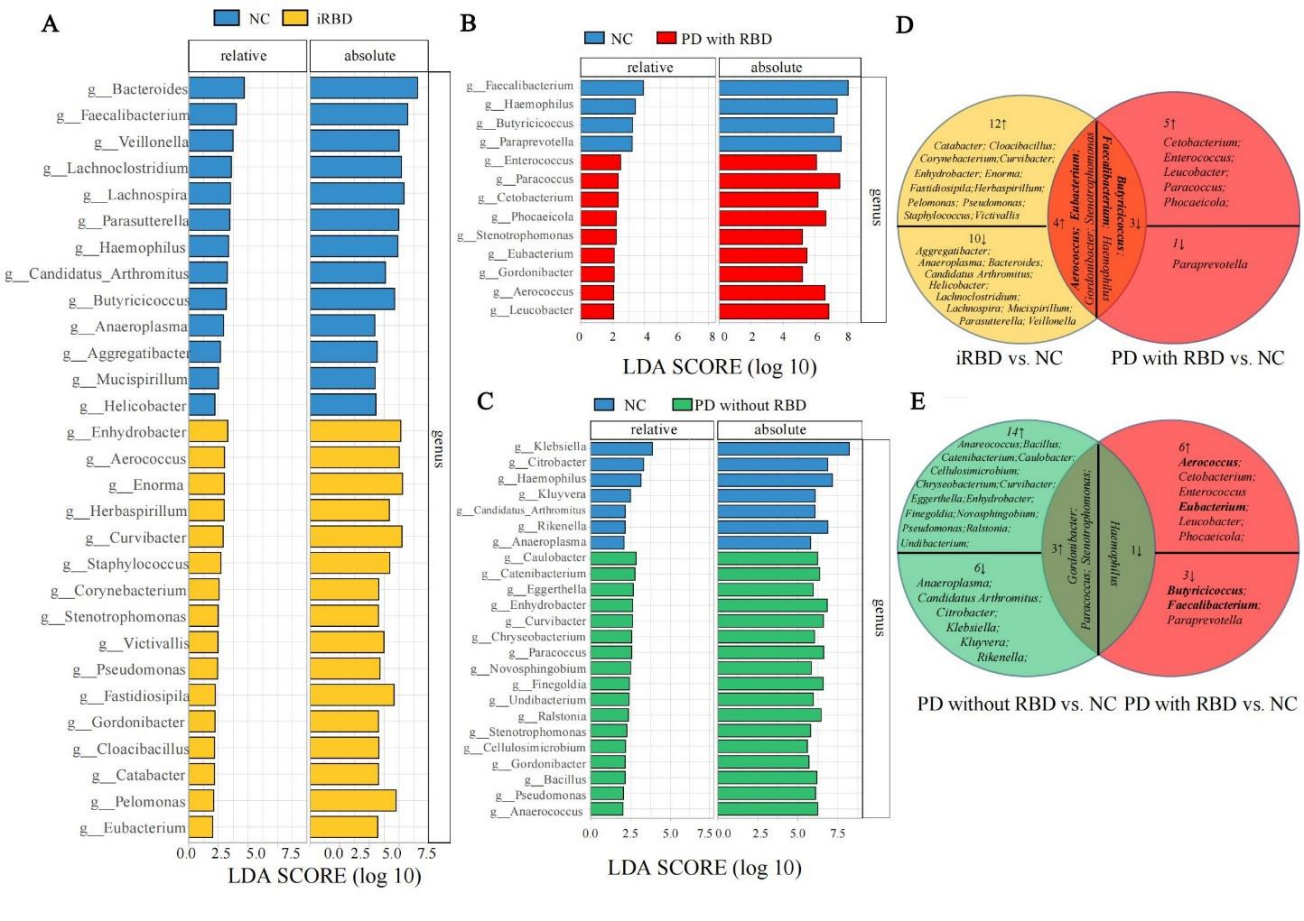
**Significant gut microbiota difference at genus level between groups in both relative and absolute data**. (**A**) A total of 29 taxa were screened out with a linear discriminant analysis (LDA) threshold score of 2.0 between iRBD and NC. (**B**) A total of 13 taxa were screened out with LDA threshold score of 2.0 between PD with RBD and NC. (**C**) A total of 24 taxa were screened out with LDA threshold score of 2.0 between PD without RBD and NC. (**D**) Flower plot demonstrating the overlapping and differential taxa in between-group comparison between iRBD versus (vs.) NC and PD with RBD vs. NC. (**E**) Flower plot demonstrating the overlapping and differential taxa in between-group comparison between PD without RBD vs. NC and PD with RBD vs. NC. NC, n = 60; iRBD, n = 35; PD without RBD, n = 64; PD with RBD, n = 30. Data were selected by Wilcoxon test with *P* < 0.05 and log 10 LDA threshold=2. Absolute abundances were analyzed by triple replicates in qPCR.

LEfSE analysis was performed in between-group comparison of microbial abundant taxa. The consistent results of relative and absolute data at genus level were studied. In the comparison between iRBD and NC, 16 genera were significantly higher abundant while 13 genera were significantly lower abundant in iRBD group (Fig. 2A). Meanwhile, in the comparison between PD with RBD and NC, 9 genera were found to be significantly higher enriched while 4 genera were significantly less abundant in PD with RBD group (Fig. 2B). In addition, in the comparison between PD without RBD and NC, 17 genera were significantly higher enriched while 7 genera were significantly less abundant in PD without RBD group (Fig. 2C). Furthermore, we focused on the overlapping genera of iRBD vs. NC and PD with RBD vs. NC (Fig. 2D), 4 genera (*Aerococcus, Eubacterium*, *Gordonibacter* and *Stenotrophomonas*) were found consistently increased while 3 genera (*Butyricicoccus, Faecalibacterium* and *Haemophilus*) were found consistently decreased in iRBD and PD with RBD group.

Next, the non-overlapping genera of PD with RBD vs. NC and PD without RBD vs. NC (Fig. 2E) were studied. 14 genera were found significantly elevated while 6 genera were significantly decreased in PD without RBD group. Besides, 6 genera were found significantly elevated while 3 genera were significantly decreased in PD with RBD group. Among them, we further noticed several distinguishable genera were consistent with the overlapping genera in Figure 2D. 2 genera (*Aerococcus* and *Eubacterium*) were elevated consistently in iRBD and PD with RBD, and they also remained distinguishable between PD with RBD and PD without RBD. Besides, 2 genera (*Butyricicoccus* and *Faecalibacterium*) were lowered consistently in iRBD and PD, and they also remained distinctive between PD with RBD and PD without RBD.

### Correlation Between Gut Microbiota and RBD-related Clinical Features

Based on the significant association of genera and RBD-related clinical features presented both in relative and absolute results, 3 significant correlations were found in the analysis of these 7 consistently changed genera in iRBD and PD with RBD (Table. 2). *Butyricicoccus* (relative coefficient = -0.19, *P* = 0.029; absolute coefficient = -0.25, *P* = 0.004) and *Faecalibacterium* (relative coefficient = -0.20, *P* = 0.025; absolute coefficient = -0.22, *P* = 0.012) were found negatively related to RBD-HK, whereas *Stenotrophomonas* was found positively related to RBD disease duration (relative coefficient = 0.38, *P* = 0.002; absolute coefficient = 0.44, *P* < 0.001) . But after ridge regression, none of them remained significant in both relative and absolute data. Only *Stenotrophomonas* presented its significance in absolute abundance.

### Predictive Functional Analysis

**Figure 3.**
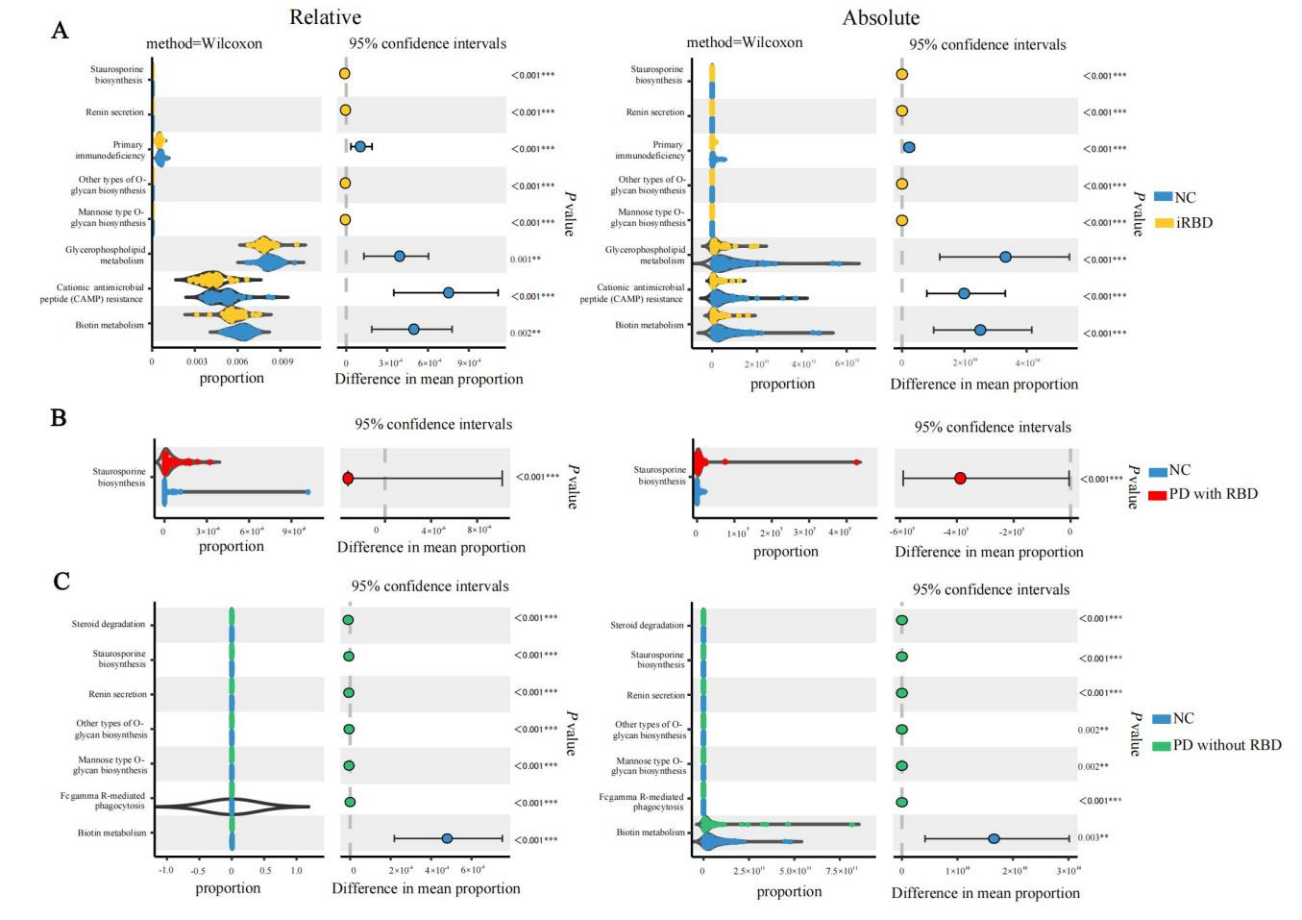
**Consistent predicted functional differences at KEGG level 3 in both relative and absolute data between groups**. (**A**) Comparison between iRBD and NC. (**B**) Comparison between PD with RBD and NC. (**C**) Comparison between PD without RBD and NC. The functional differences at KEGG level 3 were visualized by STAMP. NC, n = 60; iRBD, n = 35; PD without RBD, n = 64; PD with RBD, n = 30. Data were measured by Wilcoxon test. Absolute abundances were analyzed by triple replicates in qPCR. **P< 0.01,***P<0.001.

PICRUSt2 was performed to determine the predicted function of bacterial communities. Combing the consistent results of relative and absolute data, 4 pathways (staurosporine biosynthesis, renin secretion, mannose type O-glycan biosynthesis and other types of O-glycan biosynthesis) at KEGG level 3 were enriched in iRBD, while 4 pathways (primary immunodeficiency, glycerophospholipid metabolism, cationic antimicrobial peptide resistance and biotin metabolism) were increased in NC (Fig. 3A). In the comparison between PD with RBD and NC, staurosporine biosynthesis was increased in PD with RBD (Fig. 3B).

Besides, the increase of staurosporine biosynthesis was also distinguishable between PD without RBD and NC, while other 5 elevated pathways (steroid degradation, staurosporine, renin secretion, mannose type O-glycan biosynthesis, other types of O-glycan biosynthesis and Fc gamma R-mediated phagocytosis) and 1 decreased pathway (biotin metabolism) were observed specifically in PD without RBD vs. NC, not in PD with RBD vs. NC (Fig. 3C).

## DISCUSSION

There is a widespread debate in the field of PD pathogenesis about where the initial alpha-synuclein aggregates originate. A recent study [12] divided PD patients into brain-first (often unilaterally in the amygdala) and body-first (in the enteric or peripheral autonomic nervous system) subgroups. The body-first patients who began with gut dysbiosis were reported to have a longer prodromal RBD, and they manifested faster disease progression and more severe NMS [12,41]. Hence, the investigation of gut microbiota may provide valuable evidence for exploring the relationships between RBD and PD.

So far, we conducted a unique gut microbiota-based study in RBD and PD, which excluded the disturbance of anti-PD medications. Our study also divided PD patients into PD with RBD and PD without RBD to find distinguishable gut microbial changes. Correlation analysis was performed to confirm whether the microbial alterations were RBD clinically related. Besides, our study also met the recommendations for microbiome study of PD [42], which included absolute quantification analysis to appropriately interpret the microbial results.

In comparison of clinical features among groups, PD with RBD exhibited higher scores in Wexner, SCOPA-AUT, NMSS, total MDS-UPDRS and MDS-UPDRS III, suggesting a more severe degree of constipation, autonomic dysfunction and movement disability performance in PD with RBD. This clinical pattern was consistent with the aforementioned body-first model, which was characterized by more severe autonomic symptoms, prodromal RBD, faster progression and a higher global burden of alpha-synuclein pathology compared to brain-first PD without RBD [12].

As for the microbial structure, the indexes of alpha diversity displayed no statistical significance and this finding was in line with the result of another recently published meta-analysis [43], indicating loss of bacterial diversity is not associated with PD nor iRBD. For beta diversity analysis in unweighted and weighted UniFrac, our results demonstrated a significant difference in the overall composition among the four groups, regardless of whether the proportions were taken into consideration or not. The main statistical difference was between NC and PD with RBD. We next analyzed the enterotype distribution of each group and found iRBD, PD with RBD and PD without RBD were *Ruminococcus*-dominant while NC were *Bacteroides*-dominant. *Ruminococcus*-dominant enterotype was reported to have the lowest overall bacterial growth rate [44], the predominance of *Ruminococcus*-dominant enterotype in iRBD and two PD subgroups thus could explain the fragility of intestinal barrier and the aggravation of intestinal inflammation in the pathogenesis of PD and prodromal PD, i.e., iRBD. The restoration of intestinal homeostasis could thus ameliorate the progression of PD [45].

Next, 4 increased genera (*Aerococcus, Eubacterium*, *Gordonibacter* and *Stenotrophomonas*) and 3 decreased genera (*Butyricicoccus, Faecalibacterium* and *Haemophilus)* were found consistently changed in iRBD and PD with RBD, these seven overlapping genera might be indicative of a close relationship or phenoconversion between RBD and PD. Our findings were different from the other three previous studies [13-15], four taxa (increased *Prevotella* and *Akkermansia* in PD with RBD compared to PD without RBD, decreased *Victivallis* and *Faecalicoccus* in possible RBD) mentioned before were not statistically significant in our differential abundance comparisons and the increase of *Haemophilus* in possible RBD was even opposite to our study [15]. The explanation could be the race difference across countries, the age difference of participants in different studies [46], the inclusion of absolute abundance or the interference of anti-PD medication, etc. As another study of human intratumor microbiome also confirmed a large number of significantly differentially abundant genera with race, like in breast cancer and uterine cancer [47], we do have the reason to shed highlight on the future investigation of race diversity in microbiome study.

*Faecalibacterium* is a well-known short-chain fatty acid-producing (SCFA) genus and takes part in maintaining gut stability. A higher proportion of *Faecalibacterium* indicates a healthier state [48]. This genus was proved to be decreased in PD across five countries [49], which was consistent with our finding. Additionally, one Japanese team also confirmed the reduction of *Faecalibacterium* in iRBD [10]. It is particularly worth mentioning that *Faecalibacterium* was also reported to be associated with better sleep quality scores among bipolar disorder patients [50]. The underlying mechanism may be the production of butyrate, which has circadian rhythm regulating properties through the inhibition of pro-inflammatory cytokines [51]. *Butyricicoccus* is a type of probiotic. A previous study showed that the decrease of this genus was observed in inflammatory bowel disease, the administration of *Butyricicoccus pullicaecorum* attenuated colitis in rats and its supplementation could strengthen the epithelial barrier function by increasing the transepithelial resistance [52]. *Aerococcus* and *Stenotrophomonas* are regarded as opportunistic pathogens [53] and the elevation of *Stenotrophomonas* was observed in dysbiosis patients undergoing long-term wide-spectrum antibiotic therapy [54]. We hypothesized that the participation of the above three genera in the immune response and the modulation of intestinal permeability could be important for disease pathogenesis of RBD and PD. In line with our findings, increased *Eubacterium* was observed in another microbiome study of PD [55]. The increase of *Gordonibacter* also complied with another study, which suggested its increase in mild PD [56].

Among those 7 overlapping genera, 4 taxa (2 increased: *Aerococcus* and *Eubacterium*, 2 decreased: *Butyricicoccus* and *Faecalibacterium)* were distinctive between PD with RBD and PD without RBD. *Butyricicoccus* and *Faecalibacterium* were the two top genera which are worth mentioning, since they were also negatively correlated with RBD-related clinical features (RBD-HK), indicating their close relationship with RBD or rather RBD-specific. Noteworthily, as the reduction of *Faecalibacterium* is also compatible with the previous Japanese study and sleep disturbance in psychiatric disorder patients, which enhanced the explanatory evidence for its relation to RBD. Targeting these two genera may be an effective treatment paradigm for individuals with RBD by dietary supplementation, such as the Mediterranean diet [57].

For the functional prediction, the overlapping pathway, enriched staurosporine biosynthesis in iRBD and PD with RBD, also supported the similarity between iRBD and PD. Staurosporine stimulated SH-SY5Y neuroblastoma cells are regarded as a classic cellular model widely used in studying PD [58], the increase of staurosporine biosynthesis observed in our study might indicate the toxicity of staurosporine would active pathogenic autophagy with respect to dopaminergic cell loss [59], resulting in the pathogenesis of RBD and PD.

In conclusion, RBD has similar gut microbial changes to PD. Decreased *Butyricicoccus* and *Faecalibacterium* might be specific to RBD, and also potential hallmarks of phenoconversion of RBD to PD.

Limitations still existed in our study. First, although iRBD patients were diagnosed with PSG, the diagnosis of RBD in PD was based on RBD-HK, PSG should be included for precise diagnosis in further validating studies. Second, this study is a cross-sectional study, and a prospective longitudinal study is warranted to further confirm the role of gut microbiota alterations as a phenoconversion hallmark. Third, studies on the relevant mechanisms of specific taxa that might relate RBD to PD were rare, further experimental studies are needed to clarify their intrinsic pathogenic/protective mechanisms.

## Supplementary Materials

The Supplementary data can be found online at: www.aginganddisease.org/EN/10.14336/AD.2023.0518.


