# Supplementary material for: Relationships Between Rapid Eye Movement Sleep Behavior Disorder and Parkinson’s Disease: Indication from Gut Microbiota Alterations
Source: Aging Dis. 2024 Feb 1;15(1):357–68. doi: 10.14336/AD.2023.0518 (PMC10796088; doi:10.14336/AD.2023.0518)
Supplement: Supplementary file 1 [file AD-15-1-357-s.pdf]

SUPPLEMENTARY DATA

Relationships Between Rapid Eye Movement Sleep Behavior Disorder and Parkinson's Disease: Indication from Gut Microbiota Alterations

Pingchen Zhang, Pei Huang, Yuanyuan Li, Juanjuan Du, Ningdi Luo, Yixi He, Jin Liu, Guiying He, Shishuang Cui[1], Weishan Zhang, Gen Li, Xin Shen, Liu Jun, Shengdi Chen

© 2023. Zhang P et al. Published online at http://www.aginganddisease.org/EN/10.14336/AD.2023.0518

SUPPLEMENTARY DATA

Supplementary Table 1. Summary of the medications used among groups.

Medication type	iRBD (n=35)	PD with RBD (n=30)	PD without RBD (n=64)	NC (n=60)	Total numbers
ACEi	4	4	4	7	19
Sartan	3	3	6	11	23
CCB	0	1	2	3	6
Thiazide	2	1	4	2	9
Beta blocker	3	2	2	3	10
Levothyroxine	4	0	1	0	5
Laxative	4	1	0	1	6
Benzodiazepine	7	2	8	5	22
Alpha blocker	1	1	2	1	5
Alpha-glucosidase inhibitor	2	3	7	2	14
Sulfonylurea	1	0	1	1	3
SGLT 2 inhibitor	1	1	2	1	5

ACEi, angiotensin-converting enzyme inhibitors; CCB, calcium channel blockers; SGLT, sodium-glucose transport protein.

Supplementary Table 2. Summary of *P* value in test of normality (Shapiro-Wilk test) of demographic and clinical features and alpha diversity among groups.

Variables	iRBD (n=35)	PD with RBD (n=30)	PD without RBD (n=64)	NC (n=60)
Age(y)	＜0.001***	0.955	0.065	0.450
BMI(kg/m^2)	0.407	0.589	0.291	0.276
MMSE	＜0.001***	0.001**	＜0.001***	＜0.001***
MoCA	＜0.001***	0.417	＜0.001***	0.003**
HAMD-17	＜0.001***	＜0.001***	＜0.001***	＜0.001***
HAMA	0.041*	＜0.001***	＜0.001***	＜0.001***
Bristol	0.022*	0.026*	0.001**	＜0.001***
Wexner	0.183	0.007**	＜0.001***	＜0.001***
PD Disease duration	/	＜0.001***	＜0.001***	/
RBD Disease duration	＜0.001***	＜0.001***	/	/
RBD-HK	＜0.001***	0.001	＜0.001***	/
SCOPA-AUT	＜0.001***	0.031	＜0.001***	/
NMSS	＜0.001***	＜0.001***	＜0.001***	/
SS-16	0.281	0.544	0.127	/
PDSS	0.008	0.002	＜0.001***	/
MDS-UPDRS	/	0.037	＜0.001***	/
MDS-UPDRS III	/	0.027*	0.002**	/
Chao index	0.496	0.332	0.817	0.867
Shannon index	0.335	0.036*	0.330	0.011*
Simpson index	＜0.001***	0.003	＜0.001***	＜0.001***

BMI, body mass index; HAMA, Hamilton Anxiety Scale; HAMD-17, Hamilton Depression Scale-17 items; H-Y stage, Hoehn and Yahr stage; MDS-UPDRS, Movement Disorder Society sponsored version of the Unified Parkinson’s Disease Rating Scale; MMSE, Mini Mental State Examination; MoCA, Montreal Cognitive Assessment; NMSS, Non-Motor Symptoms Scale; RBD, rapid eye movement behavior disorder; PD, Parkinson’s disease; PDSS, Parkinson's disease sleep scale; RBD-HK, REM sleep behavior disorder questionnaire-Hong Kong; SCOPA-AUT, Scale for Outcomes in Parkinson’s disease for Autonomic Symptoms; SS-16, 16-item Sniffin' Sticks test. *$P<0.05$, **$P< 0.01$,***$P<0.001$.

© 2023. Zhang P et al. Published online at http://www.aginganddisease.org/EN/10.14336/AD.2023.0518

Supplementary Table 3. Summary of *P* value in test of homogeneity of variances (Levene test) of demographic and clinical features and alpha diversity among groups.

Variables	***P* value (among 35 iRBD, 30 PD with RBD, 64 PD without RBD and 60 NC)**
Age(y)	0.007**
BMI(kg/m^2)	0.209
MMSE	0.090
MoCA	0.025*
HAMD-17	0.001**
HAMA	0.001**
Bristol	0.002**
Wexner	0.459
PD Disease duration	＜0.001***
RBD Disease duration	0.544
RBD-HK	0.122
SCOPA-AUT	＜0.001***
NMSS	0.037*
SS-16	0.327
PDSS	0.901
MDS-UPDRS	0.353
MDS-UPDRS III	0.976
Chao index	0.496
Shannon index	0.335
Simpson index	＜0.001***

BMI, body mass index; HAMA, Hamilton Anxiety Scale; HAMD-17, Hamilton Depression Scale-17 items; H-Y stage, Hoehn and Yahr stage; MDS-UPDRS, Movement Disorder Society sponsored version of the Unified Parkinson's Disease Rating Scale; MMSE, Mini Mental State Examination; MoCA, Montreal Cognitive Assessment; NMSS, Non-Motor Symptoms Scale; RBD, rapid eye movement behavior disorder; PD, Parkinson's disease; PDSS, Parkinson's disease sleep scale; RBD-HK, REM sleep behavior disorder questionnaire-Hong Kong; SCOPA-AUT, Scale for Outcomes in Parkinson's disease for Autonomic Symptoms; SS-16, 16-item Sniffin' Sticks test. *P<0.05, **P< 0.01,***P＜0.001.

Supplementary Table 4. Summary of *P* value in test of normality (Shapiro-Wilk test) and homogeneity of variances (Levene test) of enterotype among groups.

Variables	***P* value (Levene test)**	***P* value of enterotype 1 (Shapiro-Wilk test)**	***P* value of enterotype 2 (Shapiro-Wilk test)**	***P* value of enterotype 3 (Shapiro-Wilk test)**
Bacteroides	＜0.001***	＜0.001***	0.076	0.001**
Prevotella	＜0.001***	＜0.001***	＜0.001***	＜0.001***
Ruminococcus	0.001**	＜0.001***	＜0.001***	＜0.001***

*P<0.05, **P< 0.01,***P＜0.001.

© 2023. Zhang P et al. Published online at http://www.aginganddisease.org/EN/10.14336/AD.2023.0518

SUPPLEMENTARY DATA

Supplementary Table 5. Summary of *P* value in test of normality (Shapiro-Wilk test) and homogeneity of variances (Levene test) of linear discriminant analysis (LDA) and functional prediction between iRBD and NC.

Category	Variables	Relative			Absolute		
		***P* value (Levene test)**	***P* value of NC (Shapiro-Wilk test)**	***P* value of iRBD (Shapiro-Wilk test)**	***P* value (Levene test)**	***P* value of NC (Shapiro-Wilk test)**	***P* value of iRBD (Shapiro-Wilk test)**
LDA	*Aerococcus*	0.032*	/	＜0.001***	0.088	/	＜0.001***
	Aggregatibacter	0.061	＜0.001***	＜0.001***	0.071	＜0.001***	＜0.001***
	Anaeroplasma	0.030*	＜0.001***	/	0.199	＜0.001***	/
	Bacteroides	0.030*	0.002**	＜0.001***	0.018*	＜0.001***	＜0.001***
	Butyricicoccus	0.386	＜0.001***	＜0.001***	0.692	＜0.001***	＜0.001***
	Candidatus Arthromitus	0.030*	＜0.001***	/	0.150	＜0.001***	/
	Catabacter	0.001**	＜0.001***	＜0.001***	0.682	＜0.001***	＜0.001***
	Cloacibacillus	0.005**	＜0.001***	＜0.001***	0.063	＜0.001***	＜0.001***
	Corynebacterium	0.114	＜0.001***	＜0.001***	0.633	＜0.001***	＜0.001***
	Curvibacter	0.014*	/	＜0.001***	0.003**	/	＜0.001***
	Enhydrobacter	0.016*	/	＜0.001***	0.053	/	＜0.001***
	Enorma	0.108	/	＜0.001***	0.048*	/	＜0.001***
	Eubacterium	0.440	＜0.001***	＜0.001***	0.510	＜0.001***	＜0.001***
	Faecalibacterium	0.033*	＜0.001***	＜0.001***	0.002**	＜0.001***	＜0.001***
	Fastidiosipila	0.137	＜0.001***	＜0.001***	0.084	＜0.001***	＜0.001***
	Gordonibacter	0.005**	＜0.001***	＜0.001***	0.131	＜0.001***	＜0.001***
	Haemophilus	0.038*	＜0.001***	＜0.001***	0.055	＜0.001***	＜0.001***
	Helicobacter	0.062	＜0.001***	/	0.269	＜0.001***	/
	Herbaspirillum	＜0.001***	＜0.001***	＜0.001***	0.005**	＜0.001***	＜0.001***
	Lachnoclostridium	0.339	＜0.001***	＜0.001***	0.068	＜0.001***	＜0.001***
	Lachnospira	0.033*	＜0.001***	＜0.001***	0.048	＜0.001***	＜0.001***
	Mucispirillum	0.086	＜0.001***	/	0.266	＜0.001***	/
	Parasutterella	0.103	＜0.001***	＜0.001***	0.232	＜0.001***	＜0.001***
	Pelomonas	0.019*	＜0.001***	＜0.001***	＜0.001***	＜0.001***	＜0.001***
	Pseudomonas	0.301	＜0.001***	＜0.001***	0.846	＜0.001***	0.021*
	Staphylococcus	0.042*	＜0.001***	＜0.001***	0.107	＜0.001***	＜0.001***
	Stenotrophomonas	0.047*	＜0.001***	＜0.001***	0.039*	＜0.001***	0.065
	Veillonella	0.102	＜0.001***	＜0.001***	0.033*	＜0.001***	＜0.001***
Functional prediction	Biotin metabolism	0.475	0.581	0.040*	0.024*	＜0.001***	＜0.001***
	Cationic antimicrobial peptide (CAMP) resistance	0.672	＜0.001***	0.556	0.018*	＜0.001***	＜0.001***
	Glycerophospholipid metabolism	0.782	0.475	0.014*	0.023*	＜0.001***	＜0.001***
	Mannose type O-glycan biosynthesis	0.404	＜0.001***	＜0.001***	0.653	＜0.001***	＜0.001***
	Other types of O-glycan biosynthesis	0.404	＜0.001***	＜0.001***	0.653	＜0.001***	＜0.001***
	Primary immunodeficiency	0.540	0.108	0.858	0.021*	＜0.001***	＜0.001***
	Renin secretion	0.065	＜0.001***	＜0.001***	0.092	＜0.001***	0.128
	Staurosporine biosynthesis	0.027*	＜0.001***	＜0.001***	0.016	＜0.001***	＜0.001***

LDA, linear discriminant analysis.*P<0.05, **P< 0.01,***P＜0.001.

© 2023. Zhang P et al. Published online at http://www.aginganddisease.org/EN/10.14336/AD.2023.0518

Supplementary Table 6. Summary of *P* value in test of normality (Shapiro-Wilk test) and homogeneity of variances (Levene test) of linear discriminant analysis (LDA) and functional prediction between PD with RBD and NC.

Category	Variables	Relative			Absolute		
		P value (Levene test)	*P* value of NC (Shapiro-Wilk test)	*P* value of PD with RBD (Shapiro-Wilk test)	*P* value (Levene test)	*P* value of NC (Shapiro-Wilk test)	*P* value of PD with RBD (Shapiro-Wilk test)
LDA	*Aerococcus*	0.139	/	＜0.001***	0.145	/	＜0.001***
	Butyricicoccus	0.337	＜0.001***	＜0.001***	0.107	＜0.001***	＜0.001***
	Cetobacterium	0.152	/	＜0.001***	0.091	/	＜0.001***
	Enterococcus	0.130	＜0.001***	＜0.001***	0.049*	＜0.001***	＜0.001***
	Eubacterium	0.293	＜0.001***	＜0.001***	0.061	＜0.001***	＜0.001***
	Faecalibacterium	0.052	＜0.001***	＜0.001***	0.213	＜0.001***	＜0.001***
	Gordonibacter	0.022*	＜0.001***	＜0.001***	0.115	＜0.001***	＜0.001***
	Haemophilus	0.038*	＜0.001***	＜0.001***	0.101	＜0.001***	＜0.001***
	Leucobacter	0.015*	/	＜0.001***	0.075	/	＜0.001***
	Paracoccus	0.049*	/	＜0.001***	0.083	/	＜0.001***
	Paraprevotella	0.143	＜0.001***	＜0.001***	0.194	＜0.001***	＜0.001***
	Phocaeicola	0.128	/	＜0.001***	0.109	/	＜0.001***
	Stenotrophomonas	0.180	＜0.001***	＜0.001***	0.006**	＜0.001***	0.002
Functional prediction	Staurosporine biosynthesis	0.348	＜0.001***	＜0.001***	0.068	＜0.001***	＜0.001***

LDA, linear discriminant analysis.*$P<0.05$, **$P< 0.01$,***$P<0.001$.

© 2023. Zhang P et al. Published online at http://www.aginganddisease.org/EN/10.14336/AD.2023.0518

SUPPLEMENTARY DATA

Supplementary Table 7. Summary of *P* value in test of normality (Shapiro-Wilk test) and homogeneity of variances (Levene test) of linear discriminant analysis (LDA) and functional prediction between PD without RBD and NC.

		Relative			Absolute		
**Category**	**Variables**	***P* value (Levene test)**	***P* value of NC (Shapiro-Wilk test)**	***P* value of PD without RBD (Shapiro-Wilk test)**	***P* value (Levene test)**	***P* value of NC (Shapiro-Wilk test)**	***P* value of PD without RBD (Shapiro-Wilk test)**
LDA	*Anaerococcus*	0.088	<0.001***	<0.001***	0.482	<0.001***	<0.001***
	Anaeroplasma	0.003**	<0.001***		0.082	<0.001***	
	Bacillus	0.188	<0.001***	<0.001***	0.215	<0.001***	<0.001***
	Candidatus Arthromitus	0.003**	<0.001***	/	0.051	<0.001***	/
	Catenibacterium	0.224	<0.001***	<0.001***	0.274	<0.001***	<0.001***
	Caulobacter	0.080	/	<0.001***	0.099	/	<0.001***
	Cellulosimicrobium	0.180	/	<0.001***	0.255	/	<0.001***
	Chryseobacterium	0.092	<0.001***	<0.001***	0.056	<0.001***	<0.001***
	Citrobacter	0.445	<0.001***	<0.001***	0.264	<0.001***	<0.001***
	Curvibacter	0.093	/	<0.001***	0.106	/	<0.001***
	Eggerthella	0.029 *	<0.001***	<0.001***	0.224	<0.001***	<0.001***
	Enhydrobacter	0.101	/	<0.001***	0.105	/	<0.001***
	Finegoldia	0.067	<0.001***	<0.001***	0.118	<0.001***	<0.001***
	Gordonibacter	0.012*	<0.001***	<0.001***	0.005**	<0.001***	<0.001***
	Haemophilus	0.244	<0.001***	<0.001***	0.256	<0.001***	<0.001***
	Klebsiella	0.097	<0.001***	<0.001***	0.091	<0.001***	<0.001***
	Kluyvera	0.440	<0.001***	<0.001***	0.401	<0.001***	<0.001***
	Novosphingobium	0.097	/	<0.001***	0.055	/	<0.001***
	Paracoccus	0.006**	/	<0.001***	0.057	/	<0.001***
	Pseudomonas	0.776	<0.001***	<0.001***	0.119	<0.001***	<0.001***
	Ralstonia	0.260	<0.001***	<0.001***	0.338	<0.001***	<0.001***
	Rikenella	0.038*	<0.001***	/	0.046*	<0.001***	/
	Stenotrophomonas	0.087	<0.001***	<0.001***	0.122	<0.001***	<0.001***
	Undibacterium	0.052	/	<0.001***	0.097	/	<0.001***
Functional prediction	Biotin metabolism	0.155	0.581	0.148	0.995	<0.001***	<0.001***
	Fc gamma R-mediated phagocytosis	0.175	/	<0.001***	0.233	/	<0.001***
	Mannose type O-glycan biosynthesis	0.836	<0.001***	<0.001***	0.712	<0.001***	<0.001***
	Other types of O-glycan biosynthesis	0.836	<0.001***	<0.001***	0.712	<0.001***	<0.001***
	Renin secretion	0.079	<0.001***	<0.001***	0.079	<0.001***	<0.001***
	Staurosporine biosynthesis	0.088	<0.001***	<0.001***	0.013	<0.001***	<0.001***
	Steroid degradation	0.091	<0.001***	<0.001***	0.124	<0.001***	<0.001***

LDA, linear discriminant analysis.*P<0.05, **P< 0.01,***P<0.001.

© 2023. Zhang P et al. Published online at http://www.aginganddisease.org/EN/10.14336/AD.2023.0518
